# One-Step Reduction of Graphene Oxide with Phosphorus/Silicon-Containing Compound and Its Flame Retardancy in Epoxy Resin

**DOI:** 10.3390/polym13223985

**Published:** 2021-11-18

**Authors:** Fangyi Wu, Xiaohui Bao, Jiangbo Wang

**Affiliations:** School of Materials and Chemical Engineering, Ningbo University of Technology, Ningbo 315211, China; w18758809619@163.com (F.W.); bxh19883979039@163.com (X.B.)

**Keywords:** graphene oxide, functionalization, P/Si flame retardant, epoxy resin

## Abstract

A novel graphene-based phosphorus/silicon-containing flame retardant (GO-DOPO-V) was obtained via one-step reduction of graphene oxide (GO) with phosphorus/silicon-containing compound (DOPO-V). The Fourier transform infrared (FTIR) spectroscopy, X-ray photoelectron spectrometer (XPS), Atomic force microscope (AFM) and Thermogravimetric analysis (TGA) measurements were used to confirm the structure and morphology. After incorporation of 2 wt% GO-DOPO-V, the maximum decreases of 28.8% in peak heat release rate and 15.6% in total heat release are achieved compared to that of pure epoxy resin (EP). Furthermore, TGA and Scanning electron microscopy (SEM) measurement showed that GO-DOPO-V significantly enhanced the thermal stability and residual char strength of EP. Thus, attributed to the barrier effect of GO and phosphorus/silicon layer formation by DOPO-V, GO-DOPO-V was a high-efficient flame retardant to improve the combustion behavior of EP nanocomposite.

## 1. Introduction

Because of its excellent chemical resistance, mechanical properties and low shrinkage on cure, epoxy resin (EP) has been widely used in painting, adhesives and composite applications [1,2,3,4]. However, the flame retardancy of pure EP is always not good enough to fulfill the requirement in some applications, such as semiconductor encapsulants and printed circuit boards. In recent years, many approaches have been developed to obtain halogen-free flame-retardant EP [5,6,7,8,9,10].

Phosphorus-containing compounds are one of the most promising halogen-free flame retardants, among which 9,10-dihydro-9-oxa-10-phosphaphenanthrene-10-oxide (DOPO) is a kind of environmentally-friendly flame retardant that has high thermal stability, and good oxidation and water resistance [11,12,13]. In recent years, a novel liquid compound (DOPO-V) containing DOPO and silicon was synthesized by allowing DOPO to react with vinyltrimethoxysilane (VTMS), and exhibited good compatibility with the epoxy matrix. However, a relatively high loading (10 wt%) was usually needed to achieve a good flame-retardant effect [14,15,16,17].

Graphene, as a unique two-dimensional carbon-based material, has attracted a tremendous amount of attention in various application fields [18,19,20,21,22,23]. It also could have been used as a flame retardant in the previous reports. As a barrier in polymer during combustion, graphene could slow down the heat release and block combustible fragments going into the flame area. Moreover, graphene oxide (GO) by the acid oxidation of graphite powder contains a lot of oxygen-containing groups (e.g., -OH, -COOH, epoxy) on the surface and edge, allowing the functionalization of graphene sheets via various solution reactions [24,25,26,27,28,29,30,31,32,33]. Dai et al. [34] synthesized a functional graphene (GO-MD-MP) containing POSS and DOPO groups, and added it to epoxy resin as a flame retardant. The test results show that the flame retardancy, thermal stability and mechanical properties of epoxy resin have been greatly improved. Our research group [35] has prepared a novel modified graphene (GO-PMDA) by the grafting of polysilicone to the surface of graphene oxide. The results indicate that GO-PMDA can improve the dispersion of graphene in epoxy resin. Moreover, compared with the addition of GO, the flame retardancy of epoxy resin added with GO-PMDA is better.

In this work, a novel graphene-based phosphorus/silicon-containing flame retardant (GO-DOPO-V) was synthesized via one-step reduction of GO with DOPO-V. The structure and morphology was characterized and proved by the FTIR, XPS, AFM and TGA measurements. The novel flame retardant combined flame-retardant elements phosphorus and silicone together, with which the EP incorporated not only remarkably enhanced the amount of residual char but also obtained high flame retardancy at low loading of GO-DOPO-V. Therefore, it is necessary to functionalize the graphene with DOPO-V to improve the flame retardant efficiency.

## 2. Materials and Methods

### 2.1. Materials

Graphite powders (spectrum pure), concentrated sulphuric acid (98%), phosphoric acid (85%), potassium permanganate, hydrogen peroxide (30%), 2,2′-azobisisobutyronitrile (AIBN), N,N′-dicyclohexylcarbodiimide (DCC) and tetrahydrofuran (THF) were all purchased from Alfa Aesar Chemical Reagent Co. Ltd. (Tewksbury, MA, USA). 9,10-dihydro-9-oxa-10-phosphaphenanthrene-10-oxide (DOPO) was purchased from TCI Development Co., Ltd. (Tokyo, Japan). Vinyltrimethoxysilane (VTMS), benzene (reagent grade) and ethyl alcohol (EtOH, 95%) were purchased from Sigma-Aldrich Reagent Co. Ltd. (St. Louis, MO, USA). Chloroform (CHCl_3_) and hydrochloric acid was supplied by Fisher Scientific Chemical Co. (Waltham, MA, USA). EPON 826 with an epoxy equivalent weight of 178–186 g was supplied by Hexion Specialty Chemicals Inc. (Columbus, OH, USA) and used as received. The hardener, Jeffamine D230, with an amine equivalent weight of 60 g, was supplied by Huntsman Corp. (Woodlands, TX, USA) and also used as received.

### 2.2. Synthesis of DOPO-V

DOPO (21.6 g, 0.1 mol), VTMS (14.8 g, 0.1 mol) and benzene (100 mL) were added into a three-necked flask with a mechanical stirrer, flux condenser, dropping funnel and nitrogen inlet. After the mixture was saturated with nitrogen atmosphere under vigorous mechanical stirring, the temperature was warmed to 80 °C. After the DOPO was dissolved completely, 0.1 g of AIBN, which was predissolved in 50 mL of benzene, was slowly dropped into the above reaction vessel within 2 h at 80 °C and then kept at that temperature for 24 h. After that, the products were purified by filtering. Then, benzene was removed by a rotary evaporator, yielding a colorless liquid product named DOPO-V [14].

### 2.3. Functionalization of Graphene Oxide (GO)

**Scheme 1:**
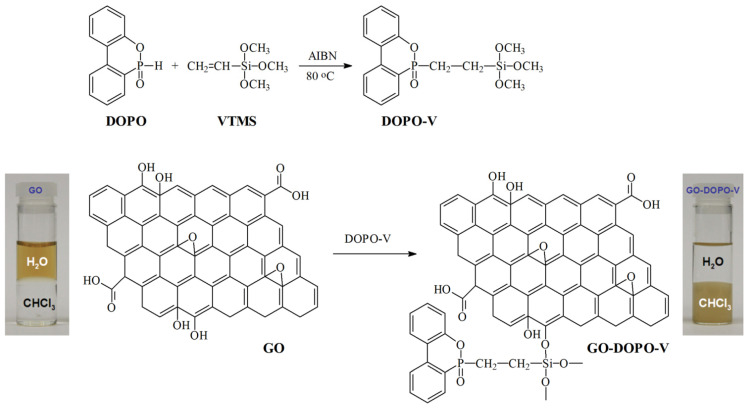
Synthetic route of GO-DOPO-V.

GO was prepared from graphite by a modified Hummers’ method [36]. As is well known, GO contains hydroxyl functional groups on their basal planes and edges, which could cause the active sites to react with silane. Briefly, the as-prepared GO (0.2 g) was first suspended in THF (200 mL) in a 500 mL three-neck flask under ultrasonication for 90 min. Subsequently, the DOPO-V (0.8 g) and DCC (0.1 g, as cat.) were introduced into the above flask, followed by ultrasonication for 30 min. With stirring, the mixture was heated to 66 °C and refluxed for 20 h under nitrogen atmosphere. Afterwards, the mixture was centrifuged and thoroughly washed with anhydrous THF to remove the residual DOPO-V. Then, the product, DOPO-V-functionalized graphene sheets (GO-DOPO-V), was dried in a vacuum at room temperature for 12 h to remove the solvent (Scheme 1).

### 2.4. Preparation of Epoxy Composite

Briefly, the EP/GO-DOPO-V composites were prepared as follows: The GO-DOPO-V (2 g) was dispersed in acetone and sonicated for 60 min to form a uniform black suspension. Then, EPON 826 (73.5 g) was added into mixture and dispersed by a mechanical stirrer for 30 min. The mixture was heated in a vacuum oven at 50 °C for 10 h to remove the solvent. After that, D230 (24.5 g) was added into mixture and stirred for 30 min. After being degassed in vacuum for 10 min to remove any trapped air, the samples were cured at 80 °C for 2 h and post cured at 135 °C for 2 h. For comparison, pure epoxy (EP), 2 wt% DOPO-V/epoxy (EP/DOPO-V) and 2 wt% GO/epoxy (EP/GO) composites were also prepared at same processing condition.

### 2.5. Characterization and Measurement

The Fourier transform infrared (FTIR) spectroscopy was tested using a Digilab Scimitar FTS-2000 IR spectrometer (Digilab Inc., Hopkinton, MA, USA) at a resolution of 2 cm^−1^ with 20 scans. The samples were mixed with potassium bromide and pressed to a disc, which was used to measure. X-ray photoelectron spectroscopy (XPS) was carried out in a Thermo Scientific ESCALAB 250Xi X-ray photoelectron spectrometer (Thermo Fisher Scientific Inc., Waltham, MA, USA) equipped with a mono-chromatic Al Kα X-ray source (1486.6 eV). AFM observation was performed on the Bruker Dimension Icon atomic force microscope (Bruker Corp., Karlsruhe, Germany) in tapping mode. The aqueous GO suspension and DMF suspension of GO-DOPO-V were spin-coated onto freshly cleared silica surfaces. Thermogravimetric analysis (TGA) measurement was carried out on a TA instrument Q5000 thermogravimetric analyzer (TA Instrument Corp., New Castle, DE, USA). The sample (about 10 mg) was heated from 50 °C to 600 °C (or 700 °C) at a 10 °C/min heating ramp rate in nitrogen atmosphere. Cone calorimeter measurement was performed on an FTT cone calorimeter (Fire Testing Technology Ltd., East Grinstead, West Sussex, UK) according to ASTM E1354 using a cone shaped heater with an incident flex set at 50 Kw/m^2^. The dimensions of each specimen was 100 × 100 × 3 mm^3^. All the measurements were repeated three times and the results were averaged. The samples were coated with a conductive gold layer and examined by scanning electron microscopy (SEM) using an FEI Quanta 200 environmental scanning electron microscope (FEI Co., Hillsboro, OR, USA).

## 3. Results and Discussion

### 3.1. Characterization of GO-DOPO-V

**Figure 1:**
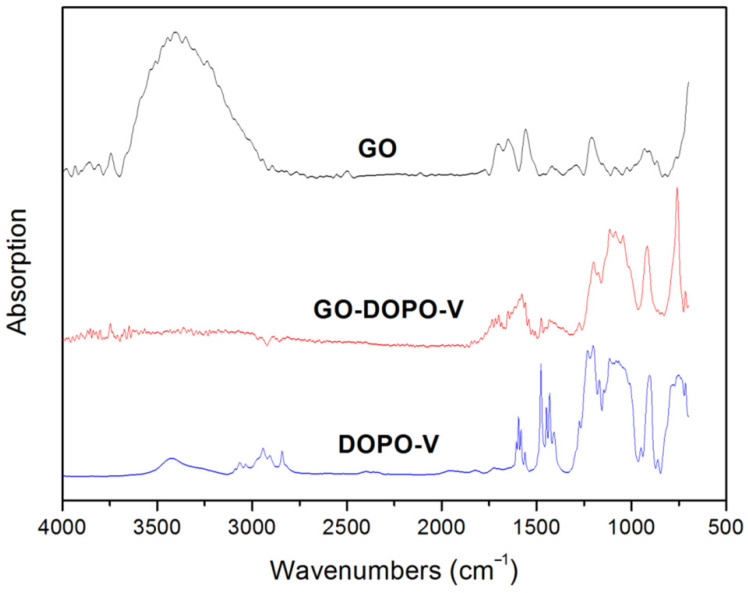
FTIR spectra of GO, DOPO-V and GO-DOPO-V.

Due to the carboxylic, epoxy, carbonyl, and hydroxide groups on its surface and edge, GO can produce stable dispersions in water with a color of light yellow after a suitable ultrasonic treatment [37,38]. As shown in Figure 1, the covalent bonding of DOPO-V onto graphene is evident by a phase transfer of the GO starting material from the water phase into the CHCl_3_ phase upon the formation of GO-DOPO-V.

The chemical structure of GO, DOPO-V and GO-DOPO-V was characterized by FTIR spectra (Figure 1). The FTIR spectra of DOPO-V showed that the characteristic peak at around 1200–1000 cm^−1^ belonged to Si-O-C and Si-O-Si structures [31,39]. The absorption peaks at 902 cm^−1^, 1274 cm^−1^ and 1595 cm^−1^ correspond to the stretching vibrations of P-O-Ph, Ph=O and P-Ph bonds, respectively. These findings verify that the DOPO-V has been successfully prepared [14]. The FTIR spectra of GO shows significant contribution from -OH and C=O chemical groups, consistent with infrared spectra of GO presented elsewhere. The strong absorption bands at about 3406 cm^−1^ originating from the stretching mode of -OH groups, indicated the existence of H_2_O and -COH within GO [40,41]. For the FTIR spectra of GO-DOPO-V, the majority of the absorption peaks that appeared in both GO and DOPO-V were observed and the peak intensities of the -OH and C=O groups decreased, which indicates that GO is grafted by DOPO-V.

**Figure 2:**
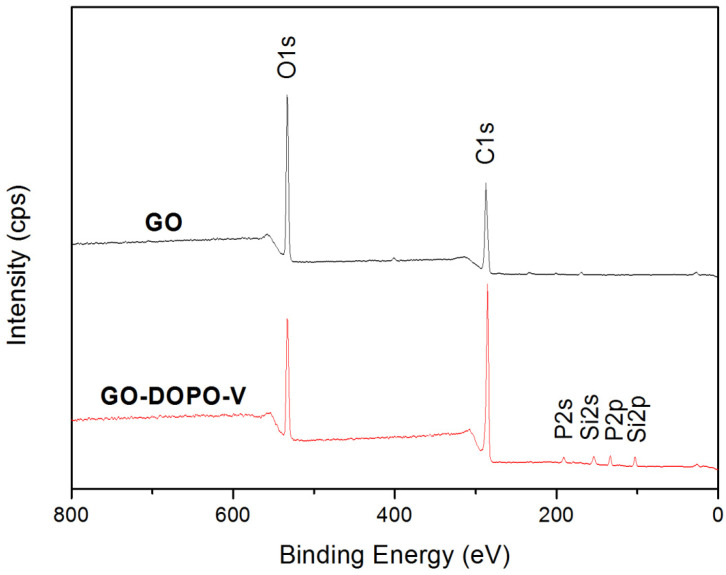
XPS survey spectra of GO and GO-DOPO-V.

The XPS spectra was used to investigate the chemical components of GO and GO-DOPO-V. As shown in Figure 2, XPS survey spectra of GO displays the presence of C1s and O1s peaks, and no peaks corresponding to other elements are detected. Thus, the appearance of the Si2p (102 eV), P2p (133 eV), Si2s (154 eV) and P2s (191 eV) peaks in the XPS survey spectra of GO-DOPO-V indicates the successfulness of the covalent bonding of DOPO-V onto GO.

**Figure 3:**
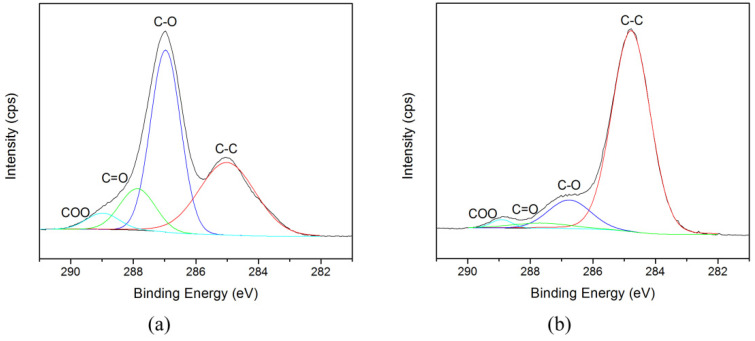
High-resolution XPS spectra of C1s for GO (**a**) and GO-DOPO-V (**b**).

The high-resolution XPS spectra of C1s is shown in Figure 3. The asymmetric shape of the C1s spectrum recorded from the unmodified GO in Figure 3a is very typical for carboneous substances consisting of graphite-like bonded carbon atoms, such as GO [42]. It exhibits the presence of four kinds of carbon in GO and GO-DOPO-V: C-C (285.0 eV), C-O (287.0 eV), C=O (287.9 eV) and COO (289.0 eV). In comparison with GO, the peaks of C-O, C=O and COO in the C1s scan of GO-DOPO-V (Figure 3b) obviously decreased, which further confirmed that GO was modified by the DOPO-V.

**Figure 4:**
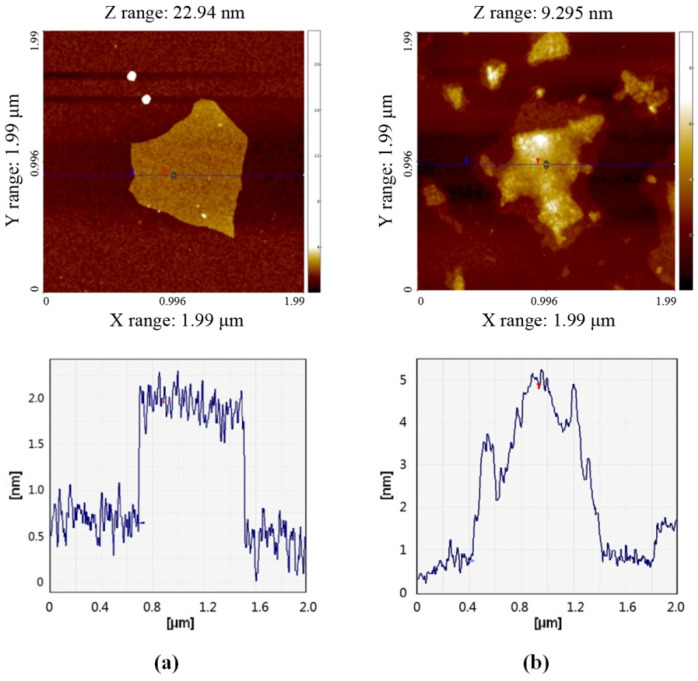
AFM images of GO (**a**) and GO-DOPO-V (**b**).

**Figure 5:**
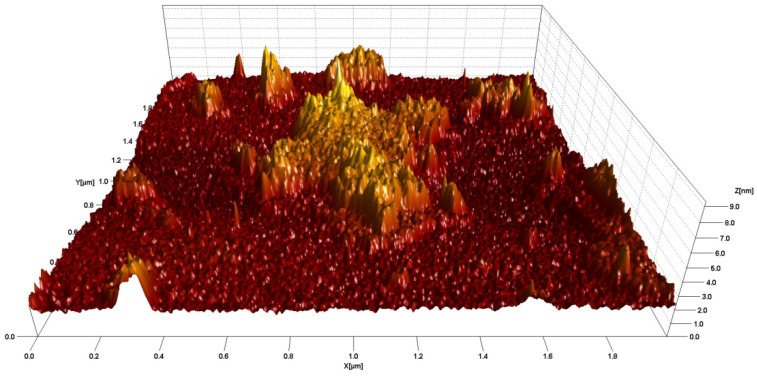
AFM images of GO-DOPO-V in the 3D view.

Figure 4 shows the tapping mode AFM images of GO and GO-DOPO-V with the height profile. After surface functionalization of GO, a layer of bright substances is observed on the graphene sheet in Figure 4b, which can be attributed to the incorporation of DOPO-V on both sides of the graphene sheet. In the appearance of graphene, as we can see, the height profile changes [33]. The grafted DOPO-V onto the surface of the GO sheet heightens its thickness to 2~4 nm in Figure 4b, compared to the thickness of GO at about 1 nm in Figure 4a. Moreover, the 3D view of GO-DOPO-V (Figure 5) reveals that the surface of GO-DOPO-V is uneven, which is probably caused by the trimethoxy groups from DOPO-V molecular. The condensation reaction between DOPO-V molecular via methoxy groups can form the building blocks of polysilicone [43,44] and, subsequently, leads to the non-uniform morphology of the surface of the GO-DOPO-V sheet.

**Figure 6:**
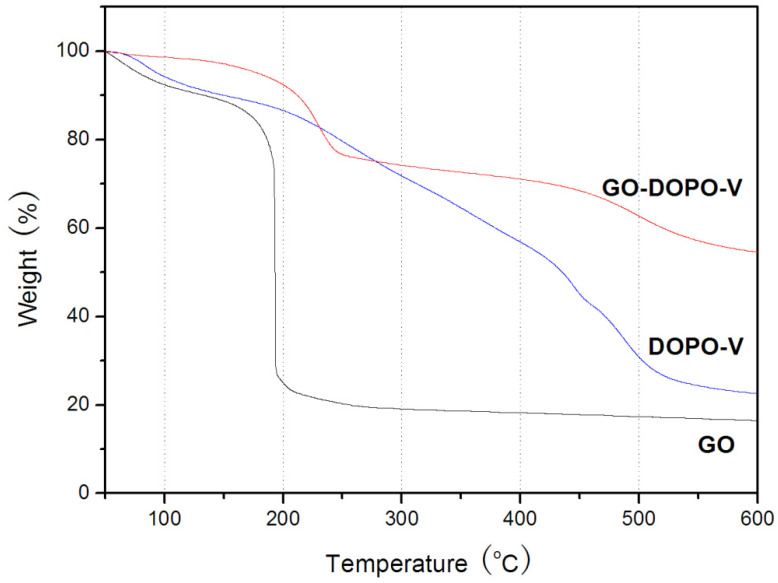
TGA curves of GO, DOPO-V and GO-DOPO-V.

The TGA curves of GO, DOPO-V and GO-DOPO-V under nitrogen atmosphere are shown in Figure 6. The temperature of 5 wt% weight loss (*T_5wt%_*) for GO is at 78.5 °C, with the maximum weight loss rate occurring at 193.1 °C. Compared with these, the temperatures of 5 wt% weight loss and maximum weight loss rate for GO-DOPO-V are 178.7 °C and 230.8 °C, respectively. It was found that reduced GO was thermally stable and subject to minor mass loss in a nitrogen atmosphere because most of the oxygen-containing groups on the surface of GO were removed in the reduction process [45]. More importantly, the amount of residue char at 600 °C also dramatically increased from GO (16.5 wt%) to GO-DOPO-V (54.6 wt%). This could be attributed to the presence of DOPO-V, which has a high gas-phase activity and condensed-phase activity (through char formation) [14,46]. Thus, GO-DOPO-V exhibits a higher thermal stability. According to the method in the literature [47], the amount of DOPO-V grafted onto the surface of the graphene was calculated, and the result of the grafting percent was 61.6%.

### 3.2. Thermal Stability

**Figure 7:**
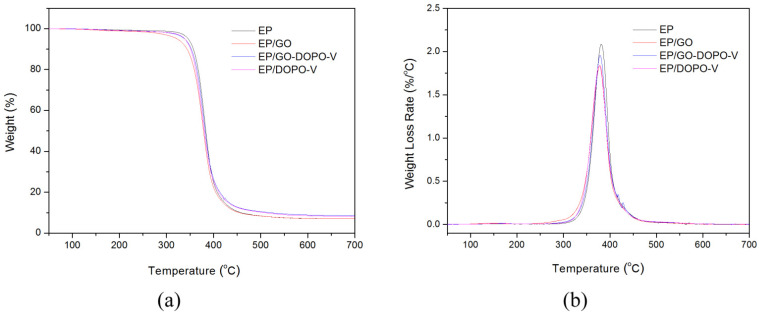
TGA (**a**) and DTG (**b**) curves of EP, EP/GO, EP/DOPO-V and EP/GO-DOPO-V.

As shown in Figure 7, the effect of GO, DOPO-V and GO-DOPO-V on the thermal stability of EP was investigated by TGA measurement, and corresponding data is summarized in Table 1. The *T_5wt%_* and *T_50wt%_* of EP/DOPO-V and EP/GO/DOPO-V exhibit similar values, but these values are still higher than those of EP/GO. Interestingly, the peak rate and *T_max_* of EP/GO-DOPO-V are both higher than those of EP/GO and EP/DOPO-V, which means EP/GO-DOPO-V has a higher temperature of main chain thermal decomposition than EP/GO and EP/DOPO-V.

Moreover, in the case of EP/GO, its residue char is lower than pure EP, since GO is thermally unstable. The addition of DOPO-V and GO-DOPO-V exhibits a reverse trend in the residue char compared with GO. In comparison with EP, the residue char of EP/DOPO-V and EP/GO-DOPO-V is increased by 1.17 wt% and 1.50 wt%, respectively. It can be concluded that since both Si and P elements have the function of promoting the formation of residue char, the incorporation of DOPO-V in GO has a marked influence on the thermal decomposition behavior of EP.

### 3.3. Flame Retardancy

**Figure 8:**
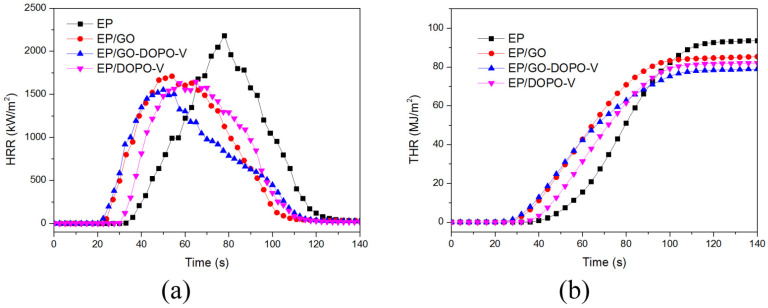
HRR (**a**) and THR (**b**) curves of EP, EP/GO, EP/DOPO-V and EP/GO-DOPO-V.

The flame retardancy of EP, EP/GO, EP/DOPO-V and EP/GO-DOPO-V is measured by cone calorimeter. Figure 8 shows the heat release rate (HRR) and total heat release (THR) curves of all of the samples. As expected, the peak heat release rate (PHRR) and THR are both decreased compared to that of pure EP, and the maximum decreases of 28.8% in PHRR and 15.6% in THR are achieved by GO-DOPO-V. When compared with GO, GO-DOPO-V exhibits greater improvement in flame retardancy of EP. The HRR and THR values of EP/GO are 1710.39 kW/m^2^ and 85.32 MJ/m^2^, respectively, while the corresponding values of EP/GO-DOPO-V are 1552.78 kW/m^2^ and 78.97 MJ/m^2^, respectively. In the HRR curves of all samples, the time corresponding to PHRR of pure EP is the longest, while the time corresponding to PHRR decreases gradually after adding various flame retardants. Among them, EP/GO-DOPO-V is the shortest. This is because the flame retardant contains phosphorus elements and unstable groups, which will induce the early degradation of EP. In the degradation process, a residual char layer will be formed to complete the flame retardant process.

**Figure 9:**
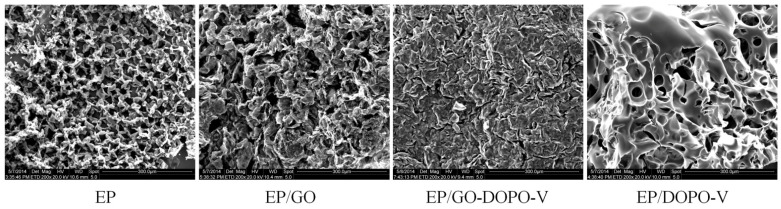
SEM images of residual char after CONE measurement.

Figure 9 shows SEM images of the residual char after CONE measurement. It can be observed that EP is a highly flammable material and its residual char shows an obviously honeycomb structure. Moreover, the char of EP/GO and EP/DOPO-V is bumpy and porous, which is caused by the gaseous products during combustion, and the volatilization of these gasses consequently leads to the formation of multi-porous interior chars [48,49]. For EP/GO-DOPO-V, homogeneous, continuous and compact residual char is formed. This can be attributed to the barrier effect of GO and the phosphorus/silicon layer formation by DOPO-V. From there comparisons, it is clear that owing to the chemical combination of GO and DOPO-V during char formation, the strength of EP/GO-DOPO-V residual char is improved, which can protect the underlying polymer and inhibit the exchange of degradation products, combustible gases and oxygen. Finally, the combustion behavior of EP is strongly enhanced, which is in accord with the results shown in TGA analysis and CONE measurement.

## 4. Conclusions

In conclusion, functionalized graphene with grafting DOPO-V was successfully synthesized through a one-step reduction method and applied to prepare the flame retardant epoxy composites. FTIR and XPS spectra show that the DOPO-V has been successfully grafted onto the surface of GO. The thickness of the GO-DOPO-V sheet is increased from about 1 nm of GO to 2~4 nm and GO-DOPO-V exhibits a higher char yield. Furthermore, the incorporation of 2 wt% GO-DOPO-V contributes an excellent thermal stability and flame retardancy to EP. From the TGA, SEM and CONE measurements, it can be concluded that, attributed to the barrier effect of GO and the phosphorus/silicon layer formation by DOPO-V, GO-DOPO-V is a highly-efficient flame retardant able to improve the combustion behavior of EP nanocomposite.

## Figures and Tables

**Table 1:** TGA data of EP, EP/GO, EP/DOPO-V and EP/GO-DOPO-V.

Sample	Temperature (°C)			Peak Rate (wt%/°C)	Residue Char (wt%)
	*T_5wt%_*	*T_50wt%_*	*T_max_*		
EP	346.0	383.5	381.4	2.09	7.02
EP/GO	322.5	378.6	378.2	1.84	6.95
EP/DOPO-V	338.7	380.2	376.8	1.84	8.19
EP/GO-DOPO-V	338.4	381.9	379.0	1.96	8.52

## Data Availability

The data used to support the findings of this study are available from the corresponding author upon request.
